# Secular trends in new childhood epidemics: insights from evolutionary medicine

**DOI:** 10.1186/1741-7015-11-226

**Published:** 2013-10-21

**Authors:** Martin Brüne, Ze´ev Hochberg

**Affiliations:** 1 LWL University Hospital, Division of Cognitive Neuropsychiatry and Psychiatric Preventive Medicine, Ruhr-University Bochum, Alexandrinenstra?e 1, 44791, Bochum, Germany; 2 Rappaport Family Faculty of Medicine, Technion-Israel Institute of Technology, Haifa 30196, Israel

**Keywords:** Pediatric endocrinology, Life history, Hygiene, Pathogen exposure, Autoimmune diseases, Evolutionary mismatch, Psycho-biological development, Psychopathology, Obesity, Diabetes

## Abstract

In the last few decades, pediatric medicine has observed a dramatic increase in the prevalence of hitherto rare illnesses, among which obesity, diabetes, allergies and other autoimmune diseases stand out. In addition, secular trends towards earlier onset of puberty and sexual activity contribute to the psychological problems of youth and adolescents. All this has occurred in spite of the improved health care provision for children, yet traditional concepts of medicine have failed to explain these new ?epidemics?.

A recent conference and science school of the European Society of Paediatric Endocrinology (ESPE) in Acre, Israel, has taken up this challenge. Experts across disciplines including medicine, anthropology and developmental psychology discussed potential causes of childhood ill-health from an evolutionary point-of-view. Seen from an evolutionary vantage point, the ?epidemics? of childhood obesity, diabetes and psychological dysfunction appear, in part, to be related to a mismatch between ancestral adaptations and novel environmental contingencies. These include changing exposures to pathogens, which impact on the function of the immune system, as well as changing patterns of parenting, which influence the timing of puberty and the risk for developing psychopathology.

## Introduction

For a few decades, pediatricians have begun to face ?new epidemics? affecting children?s physical and mental health. Rather than fighting novel infectious diseases as the leading cause of childhood mortality throughout human history, new challenges for child healthcare have arisen from overweight, changes in growth patterns, earlier sexual activity, as well as from other secular transformations pertaining to the conditions of up-bringing. Ironically, in spite of improved access to healthcare and treatment options for many childhood diseases, clinicians have observed a dramatic increase in chronic diseases, including, among many, childhood obesity, diabetes and autoimmune diseases, particularly since the 1980s [1,2]. There is also a clear secular trend towards increased height and weight in most developed countries, which affects the timing of puberty and onset of sexual activity [3]. These changes have occurred too rapidly as to invoke genetic modification as the causal factor. Instead, it is much more likely that epigenetic regulation of growth and maturational patterns, that is, developmental plasticity, has been decisive in this regard, a view that is strongly supported by comparisons between populations that differ in nutritional supply, exposure to pathogens and socio-economic status [4]. However, until quite recently, the specific environmental contingencies interacting with genes involved in growth and development have remained elusive.

In May 2013, a conference of the European Society for Paediatric Endocrinology (ESPE) on Child Health and Human Evolution in Acre, Israel, took up these open questions from a scientific angle that has become more and more influential in the recent past to explain gene-environment interaction with respect to health issues, namely Evolutionary Medicine.

Evolutionary Medicine uses the reconstruction of *Homo sapiens*? evolutionary past that brought about physical and psychological adaptations to quite a diverse spectrum of Environments of Evolutionary Adaptedness (EEAs). Thus, it offers a great deal of insight into present-day health issues, providing clinical clues for how the conditions of modern life deviate from ancestral ones, and why adaptations to past environments can have detrimental effects in contemporary settings. In this essay, based on the lectures and discussions at the Acre Conference, we highlight some of the most intriguing aspects of the secular trend and developmental plasticity with respect to child health and discuss potential consequences for healthcare and disease prevention.

### Childhood obesity

Secular trends in childhood obesity represent a growing health issue in the developed countries with profound impact on adult health [4]. Over the past 130 years or so, the body mass index (BMI) has constantly increased in developed countries, with figures of obesity having constantly risen, particularly since the 1980s. The prevalence of childhood obesity is currently below 5 percent in African countries, with figures of around 20 percent in Europe and over 30 percent in North America [5]. It is non-existent in traditional societies of hunter-gatherers, which have served as models of Paleolithic human societies. Another simple method of determining body fat deposition is the examination of the thickness of the skin over the triceps muscle, where a German study found that while the thickness of the triceps skinfold increased dramatically from 8.3 mm to 27.1 mm in 30 years, from 1975 to 2005, the trend started as early as the early 1900s [6,7].

Among the most ?obesogenic? factors are increasing energy consumption, mainly foods rich in fat and sugar, and decreasing physical activity with the adoption of a more sedentary lifestyle - a whole mark of contemporary EEA. Obviously, the oversupply of energy and the lack of physical activity and exercise is an example of an evolutionary mismatch. Over tens of thousands of years of the Paleolithic era, humans lived in conditions where the availability of food could change dramatically. Food scarcity and famine probably occurred much more often than times of food abundance. According to the ?thrifty gene? hypothesis [8], humans have, therefore, evolved mechanisms for maximizing the extraction of energy from food, and store energy in fat depots, which now turn into a disadvantage with regards to health. Transition to farming was associated with frequent famines that hindered the adjustment of the genome to the newly established mostly-carbohydrate diet. So, arguably, a mismatch between a Paleolithic genome and a Holocene diet is key to the obesity epidemic we observe today.

Another factor that may profoundly impact the risk of developing overweight and obesity is related to the intrauterine environment. ?Fetal programming? suggests that intrauterine conditions imprint the fetus? postnatal development with long-lasting effects extending into adult life [9]. This makes sense from an evolutionary perspective, because an organism that is adaptively prepared before birth for optimally dealing with conditions after birth has a greater chance for survival (and reproduction). Excessive maternal intake of calories during pregnancy, for instance, may influence the development of the fetus? first fat lobules *in utero*. Indeed, the average weight gain during pregnancy has increased by 2 kg in 20 years time [10]. Fetal programming has actually been considered as a causal factor for an amazingly broad range of trends in physical and psychological disorders, including cardiovascular disease [11], diabetes [12], cancer [13], respiratory disease [14], obesity [15] and attention deficit hyperactivity disorder [16], to name just a few.

Finally, assortative mating, an evolved pattern of how individuals choose their sexual partners, which suggests that partner choice is based, in part, on physical similarity, may impact the risk of obesity, because of the potential accumulation of ?thrifty genes? or genes involved in the regulation of fat deposition or satiety. In summary, childhood obesity illustrates at several levels how adaptive mechanisms that evolved in the ancestral past can contribute to the development of new health hazards. Related to this, diabetes is a pressing health issue with some overlapping predispositions with obesity, but additional evolutionary factors that might be equally relevant.

### Childhood diabetes

Childhood diabetes type I is another example illustrating secular trends towards increased risk for specific metabolic disorders [17]. Type-I diabetes is an autoimmune disease with a genetic risk related to the make-up of the HLA system. It is believed that the manifestation of type-I diabetes is driven by an exogenous factor, most likely an infectious agent. According to a Finnish study, the incidence of type-I diabetes among children younger than 15 years has increased from 12 per 100,000 in the early 1950s to 65 per 100,000 in 2006 [18]. The causal factors underlying this dramatic increase have been obscure, but Evolutionary Medicine offers some possible explanations that may independently contribute to this growing child health problem.

One plausible explanation relates to the so-called ?hygiene hypothesis?, suggesting that the decrease in diversity of pathogens children are exposed to has led to an increase in autoimmune diseases, including type-I diabetes [19]. In support of this hypothesis, a comparison of children living in the region of Karelia, Russia, with children from Finland who do not differ in terms of their genetic make-up demonstrates that the exposure to pathogenic agents is much higher in Karelia. Karelian children have a 15-fold higher prevalence of *Helicobacter pylori* antibodies, a 5-fold higher frequency of *Toxoplasma gondii* antibodies, a 12-fold higher prevalence of hepatitis A antibodies, and a 20% higher frequency of Coxsackie virus B4 antibodies as compared to Finnish children. In contrast, the figures for autoimmune diseases point in the opposite direction, where Karelian children have a six-fold lower incidence rate of type-I diabetes, and similar figures have been obtained for celiac disease, autoimmune thyreoiditis, and allergen-specific IgE responses [20]. These differences could, in part, be related to differences in gut microbiome, showing that healthy children have a greater diversity of intestinal bacteria than children with autoimmune diseases [21]. This example suggests that ancient adaptation to past pathogenic environments may now increase the vulnerability of children (and adults) for developing autoimmune diseases, including childhood diabetes, asthma and other allergic responses.

Another possible factor accounting for the increase of type-I diabetes in children resides in changing habits of nurturing and feeding. Early exposure to cow milk induces an immune response to bovine insulin, which may later ?spark over? to attack endogenous insulin in individuals who lack the tolerance of oral ingestion of bovine insulin. In fact, IgG antibodies against bovine insulin are elevated in formula-fed infants at three months of age compared to breast-fed infants [22]. Again, this example indicates that a mismatch between past adaptations and current environmental contingencies can contribute to a rise of ?new epidemics?, which, ironically, hit the most economically developed countries in Europe and North America harder due to the better socio-economic status. It can be expected, though, that similar health issues will occur in the less economically developed countries in Asia, South America and Africa once their socio-economic conditions have improved, unless preventive measures, such as the promotion of breast-feeding, have become effective. In fact, a recent review reports that breast-feeding was associated with a reduction in the risk of acute inflammatory diseases, such as acute otitis media, gastroenteritis, lower respiratory tract infections, but also with reduced numbers of autoimmune diseases including atopic dermatitis and asthma. There was also an association with a reduction in the risk of developing obesity, type 1 and 2 diabetes, childhood leukemia, sudden infant death syndrome (SIDS) and necrotizing enterocolitis [23]. 

### Psychological development

Secular transitions have not spared family structures and parenting behavior, but without evolutionary insights it could not be fully understood why the conditions of one?s upbringing have such a great impact on the psychosexual development of adolescents [24]. As early as in the 1980s, it was observed that adolescent girls who grew up without their fathers show early expression of sexual interest and assumption of sexual activity, negative attitudes toward males, and poor ability to establish long-term relationships with one male [25]. The early family environment does not only shape individuals? psychological development in terms of attachment styles, formation of trusting relationships versus the development of mistrustful ?inner working models?, but the family environment also impacts the timing of puberty, sexual activity and investment in partners and a woman?s own children. Parental warmth and emotional care has been shown to slow down pubertal development, whereas harsh and rejecting parenting styles lead to an acceleration of pubertal development (reviewed in [26]). In fact, abuse, marital conflict and lack of parental supportiveness contribute to an earlier onset of puberty in girls, as do insecure attachments and absence of the father or severe paternal psychopathology. Intriguingly, these factors also contribute to greater sexual risk-taking in adolescent girls.

In addition to these environmental factors influencing the psycho-biological development in girls, there is evidence for gene-environment interaction, suggesting that the quality of maternal care interacts with polymorphic variation of estrogen receptor-coding genes in ways that support the assumption of ?differential susceptibility? [27]. Put another way, girls carrying the GG variant of the estrogen receptor-alpha (*ESR1*) gene who grow up with less sensitive mothers are younger at menarche than AA carriers, whereas GG carriers raised by sensitive mothers experience menarche later than carriers of the AA genotype.

In a psychopathological perspective, individuals with borderline personality disorders (BPD) follow a developmental pathway associated with sexual risk-taking behavior, associated with frequent disruption of close relationships (including partnerships) and reduced investment in their own children [28]. Beyond its conceptualization as a pathology, the evolutionary view of BPD suggests that patients? behavior makes sense in a developmental perspective, as many patients (the majority of which are female) have encountered adversity during early developmental stages, including neglect, abuse and family disruption, leading to an insecure reproductive strategy.

### Outlook

Over eons, humans have adapted genetically and epigenetically to environmental contingencies, which in many ways are dissimilar to contemporary ones. Evolutionary Medicine is an emerging field that can help clarify hitherto incompletely understood health issues, including the ?new epidemics? of childhood that arise from mismatches between genes and environment [29]. Living in diverse environments that range from the tropics to the arctic, and from deserts to mountain tops, humans evolved to provide the greatest possible physiological plasticity and behavioral flexibility to changing micro- and macro-environments.

A broad range of diseases that were until recently considered to be rare have now exploded in incidence and prevalence rates, at least in the developed countries - with the prospect that the situation will be similar in the developing world, once the socio-economic status has risen to a similar level. This is, of course, not to argue that for these reasons developing countries should be precluded from improved health care, an increase in income, education and welfare - quite differently, it is rather an opportunity to prevent the developing countries from repeating the unfortunate sequence that has inflicted developed countries.

Evidence suggests that for European countries the childhood obesity and diabetes epidemics have slowed down and might have come to a halt in the last 10 years or so (for example, [30]). It is not clear which factors have contributed to this, but there is at least good reason to suggest that preventive health care measures, for example, dietary adjustments, changes in attitude towards breast-feeding, programs to increase physical activity among children and adolescents, approaching that of our ancestors, can exert positive effects on children?s health, and adults? alike.

*Homo sapiens* has evolved a distinct life-history which includes a relatively short infancy, a prolonged childhood and the emergence of adolescence as a novel developmental stage not seen in other primates. The evolved function of adolescence is most likely associated with the growing need of acquiring social skills that are necessary to successfully prepare and adopt adult social roles.

Taken together, it seems that our evolved life history patterns have come at a cost -?tradeoffs? in evolutionary jargon - which has brought about an increased risk for disruption of normal developmental pathways, where the cost becomes greater as modern environmental contingencies deviate from ancestral ones.

Insights from Evolutionary Medicine can help us understand, cure and prevent physical and psychological disease and disorders in many ways. We believe this is the way medicine has to go in the future at all levels, from research to practice. Evolutionary Medicine is coming of age.

## Competing interests

The authors declare that they have no competing interests.

## Authors? information

MB is Professor of Cognitive Neuropsychiatry and Psychiatric Preventive Medicine and the author of the *Textbook of Evolutionary Psychiatry. The Origins of Psychopathology*, Oxford University Press, 2008.

ZH is Professor of Pediatrics and Endocrinology and the author of *Evo-Devo of Child Growth*, Wiley, 2012. 
